# Reduced efficacy of food-chain biocides and preservatives against ciprofloxacin-resistant *Salmonella* Typhimurium

**DOI:** 10.1007/s00253-026-13973-0

**Published:** 2026-07-28

**Authors:** Raúl Campillo, Ivo García-Penas, Celia Pardos, Patricia Gimeno, Daniel Berdejo, Rafael Pagán, Diego García-Gonzalo

**Affiliations:** Departamento de Producción Animal y Ciencia de los Alimentos, Facultad de Veterinaria, Instituto Agroalimentario de Aragón-IA2 (Universidad de Zaragoza-CITA), C/Miguel Servet 177, 50013 Zaragoza, Spain

**Keywords:** AMR, Genetic changes, Preservatives, Disinfectants, Food preservation, Virulence

## Abstract

**Abstract:**

A wide range of microbial prevention and control strategies are applied along the agri-food chain to combat the transmission of foodborne pathogens to the final consumer. These strategies range from traditional thermal treatments to emerging technologies as well as the use of food chain biocides to reduce microbial loads on raw materials and food contact surfaces, and preservatives for the preservation of feed and food products. However, bacteria have demonstrated a remarkable ability to adapt to and overcome these food-related stresses, often resulting in reduced susceptibility to other seemingly unrelated agents through cross-protection mechanisms. In this study, we aimed to evaluate the outcomes of ciprofloxacin (CIP) antimicrobial resistance (AMR) in *Salmonella* Typhimurium and its potential relationship with cross-protection to food-chain biocides and preservatives, as well as the ability of the resistant variants (RVs) to retain their virulence. The CIP-RVs showed heterogeneous and compound-dependent responses to the tested food-chain antimicrobial compounds. All five RVs displayed increased tolerance to at least one compound, but cross-protection was not uniform across strains or treatments. Notably, SeT_RV1_ displayed reduced susceptibility to four of the six agents tested, and derivative strains carrying SeT_RV1_-derived alleles supported an important contribution of *ramR*, *cyaA*, and especially *rpoD* to this broad phenotype. Moreover, several RVs displayed *Galleria mellonella* pathogenicity comparable to that of the parental strain, despite occasional reductions or dose-dependent effects, suggesting that reduced susceptibility to food-chain antimicrobial compounds may occur without a major loss of virulence-associated traits in this model.

**Key points:**

• *CIP-resistant S. Typhimurium variants can show reduced chemical susceptibility*

• *Cross-protection is associated with global regulation and stress response mutations*

• *CIP-RVs showed divergent pathogenicity patterns in the Galleria model*

**Supplementary Information:**

The online version contains supplementary material available at https://doi.org/10.1007/s00253-026-13973-0.

## Introduction

From farm to fork, the agri-food chain relies on multiple strategies to prevent, limit, or eliminate foodborne pathogens and to reduce their transmission to the consumer (Shafiur 2007). These measures include physical technologies aimed at bacterial inactivation such as heat treatments, as well as non-thermal alternatives already implemented or still under development (e.g., pulsed electric fields, ultraviolet irradiation, high hydrostatic pressure) (Barbosa-Cánovas et al. 2004; White et al. 2025), as well as growth-limiting approaches such as refrigeration or lyophilization.

In addition to these strategies, chemical antimicrobial compounds are extensively applied along the food chain. In food-processing environments, food-chain biocides are used for hygiene and sanitation of raw materials and food-contact surfaces (e.g., sodium hypochlorite, peracetic acid, and tertiary amines). In food and feed, preservatives are incorporated to inhibit microbial growth (e.g., sodium chloride and lactic acid), as well as plant-derived antimicrobial compounds such as carvacrol, which may also contribute to organoleptic properties (Ramesh et al. 2026; Rodríguez-Melcón et al. 2017). Depending on concentration, exposure time, and environmental conditions, many of these compounds can exert either bacteriostatic or bactericidal effects. Together, they form an essential, multi-layered barrier for food safety, but they can also impose selective pressures that favor the emergence of adapted bacterial subpopulations (Giacometti et al. 2021).

Antimicrobial resistance (AMR) remains a major global concern and a clear example of bacterial adaptation driven by selective pressures exerted by antibiotics used in humans and food-producing animals. Fluoroquinolones, such as ciprofloxacin (CIP), are considered critically important antimicrobials, and increasing trends in CIP resistance have been reported in poultry-associated serovars isolated from humans (World Health Organization 2024). In 2023, *Salmonella* Typhimurium was the second most common serovar among human *Salmonella* infections (European Food Safety Authority and European Centre for Disease Prevention and Control 2025). Beyond treatment failure, AMR may also have broader phenotypic consequences that influence survival or growth under food-related stresses.

Adaptation to one stressor can alter susceptibility to other apparently unrelated stresses, a phenomenon described as *cross-resistance*, *-adaptation*, or *-protection* (for simplicity, in this work we use the term *cross-protection*). This concept is well recognized in the direction from biocide tolerance to antimicrobial cross-resistance (Coombs et al. 2023), and it has also been reported in *S.* Typhimurium variants tolerant to food-related stressors (Guillén et al. 2025; Pagán et al. 2024a, 2024b; Wu-Chen et al. 2023). However, the reciprocal direction (i.e., whether AMR can increase tolerance to food-related antimicrobial strategies) remains comparatively underexplored, and robust experimental evidence is still limited. Several studies have supported such associations for physical stressors, mostly against heat treatments (Campillo et al. 2025; Campillo et al. 2026a﻿; Collis and Grigg 1989﻿; de Zuazo et al. 2026﻿; Søballe and Poole 2000﻿; Tavares da Silva et al. 2025). For chemical compounds used in food preservation, the available evidence is also limited, although recent studies have reported altered susceptibility to preservatives such as CAR in AMR *S.* Typhimurium variants (García-Penas et al. 2026a; Tavares da Silva et al. 2025).

To address this gap, we previously evolved a *S.* Typhimurium parental strain (SeT) through adaptive laboratory evolution (ALE) under increasing CIP concentrations, isolating five CIP-resistant variants (RVs). In one of these RVs, CIP resistance was positively associated with decreased susceptibility to heat treatments (Campillo et al. 2025). Here, we extend this approach to farm-to-fork-related antimicrobial compounds used for microbial control in the food industry, including food-chain biocides and preservatives. In addition, we evaluate whether these RVs retain virulence, since the AMR-virulence relationship can range from neutral to reduced or even enhanced pathogenic potential (Campillo et al. 2025﻿; Cepas and Soto 2020).

Therefore, the central question addressed in this study was whether CIP-driven evolution in *S.* Typhimurium can generate RVs with altered susceptibility to food-chain antimicrobial compounds while retaining virulence-associated traits, and whether defined mutations from a broadly tolerant variant contribute to these phenotypes. To address this question, we first compared the survival of five CIP-RVs (SeT_RV1_-SeT_RV5_) against selected food-chain biocides and food/feed preservatives. We then evaluated the contribution of key SeT_RV1_ mutations (*rpoD*, *cyaA*, and *ramR*) using engineered SeT derivative strains carrying SeT_RV1_-derived alleles, alone or in combination. Finally, we assessed whether the CIP-RVs displayed altered pathogenicity in the *Galleria mellonella* infection model.

## Materials and methods

### Microorganisms, growth conditions, and reagents

We used five CIP-RVs previously isolated in our lab (SeT_RV1_-SeT_RV5_) and genotypically characterized by whole-genome sequencing (Campillo et al. 2025). The parental strain used in this study was *Salmonella enterica* subsp. *enterica* serovar Typhimurium str. LT2 (SeT), obtained from the Spanish Type Culture Collection (CECT 722). SeT is a laboratory reference strain and was not isolated from a specific food industry, animal feed, or agri-food environment in the context of this study (NCBI accession: NC_003197.2). The CIP-RVs were obtained after subjecting SeT to ALE experiments based on increasing doses of CIP during bacterial growth (García-Penas et al. 2026b). Briefly, the RVs harbor mutations in genes associated with transcriptional regulation (*ramR*, *soxR*), RNA polymerase subunits (*rpoD*, *rpoC*), DNA topology (*gyrA*, *gyrB*), and central metabolic or signaling pathways (*cyaA*, *thrS*, *purB*), as well as mutations in intergenic regions affecting regulatory loci. Details of each strain’s mutations are summarized in Table 1.

**Table 1:** Genetic variations in ciprofloxacin-resistant variants (SeT_RV1_-SeT_RV5_) relative to the parental strain *Salmonella enterica* subsp. *enterica* serovar Typhimurium str. LT2 (SeT). Adapted from Campillo et al. (2025)

Strain	Gene	Mutation type	Predicted effect
**SeT**_**RV1**_	*ramR*	SNV	Amino acid substitution
	*rpoD*	Insertion	In-frame insertion
	*cyaA*	Deletion	Frameshift (likely loss of function)
**SeT**_**RV2**_	*thrS*	Deletion	In-frame deletion
	*soxR*	Deletion	In-frame deletion
**SeT**_**RV3**_	*purB*	Insertion	Frameshift (likely loss of function)
	*ychM*	SNV	Upstream variant (possible regulatory effect)
	*gyrA*	SNV	Amino acid substitution
	*soxR*	Deletion	Frameshift (likely loss of function)
**SeT**_**RV4**_	*ramR*	Deletion	Frameshift (likely loss of function)
	*gyrA*	SNV	Amino acid substitution
	*rpoC*	SNV	Amino acid substitution
	*soxR*	Deletion	Upstream variant (possible regulatory effect)
**SeT**_**RV5**_	*ramR*	Insertion	Frameshift (likely loss of function)
	*ramA*	Deletion	Upstream variant (possible regulatory effect)
	Prophage protein	SNV	Silent mutation
	Prophage protein	SNV	Silent mutation
	*gyrB*	SNV	Amino acid substitution

Genetic variations between SeT and the reference genome are omitted

*SNV*, single-nucleotide variation; *Frameshift*, mutation shifts codon reading frame due to mutation; *Silent*, mutation does not alter the encoded amino acid

This study also included SeT derivative strains carrying SeT_RV1_-derived alleles (i.e., the SeT_RV1_ allele sequence at each locus, relative to SeT) either individually (SeT_*rpoD*_, SeT_*cyaA*_, and SeT_*ramR*_) or in combination (SeT_*rpoD*__*/*__*cyaA*_, SeT_*rpoD*__*/*__*ramR*_, and SeT_*cyaA*__*/*__*ramR*_). These alleles were previously shown to contribute to tolerance to thermal treatments (Campillo et al. 2025). The derivative strains were generated by scarless targeted genome editing in the SeT genetic background, as described elsewhere (Campillo et al. 2025, 2026b), enabling assessment of the contribution of the indicated SeT_RV1_ mutations to specific phenotypes.

The term *AMR-related genes* refers here to chromosomal genes involved in pathways associated with antimicrobial susceptibility or resistance, including efflux regulation, DNA topology, transcriptional regulation, and stress responses, rather than to acquired antibiotic-resistance marker genes. The evolved RVs did not share an identical set of mutations, but each displayed a specific mutational profile, as summarized in Table 1. Therefore, phenotypes observed in the RVs were interpreted in the context of their complete genetic backgrounds. In contrast, the engineered SeT derivative strains carried only the indicated SeT_RV1_-derived alleles (*ramR*, *cyaA*, and/or *rpoD*) in the SeT background and were generated by scarless genome editing without introducing or retaining additional antibiotic-resistance markers.

Working cultures were prepared from cryovials stored at − 80 °C by streaking onto tryptone soya agar supplemented with 0.6% (*w/v*) yeast extract (TSAYE) and incubating overnight at 37 °C. A single colony was inoculated into 5 mL of cation-adjusted Mueller–Hinton broth (MHB) and incubated overnight at 37 °C with orbital shaking (130 rpm) under aerobic conditions. These cultures were used to inoculate fresh MHB (10 mL) to an initial concentration of approximately 10^6^ CFU/mL and incubated at 37 °C with shaking (130 rpm) for 24 h until reaching stationary phase (~ 3 × 10^9^ CFU/mL). Cell concentrations were determined by serial dilution in phosphate-buffered saline (Sigma-Aldrich; PBS) followed by spread plating on TSAYE. All strains were stored at − 80 °C in cryovials containing glycerol (20% *v/v*) during the course of this investigation.

### Cross-protection against farm-to-fork antimicrobial compounds

To assess whether CIP resistance is associated with altered tolerance to commonly used farm-to-fork antimicrobial compounds, we selected substances with distinct chemical properties. As food-chain biocides, we tested sodium hypochlorite (13% *w/v* active chlorine, Sigma-Aldrich, St. Louis, USA; NaOCl), peracetic acid (39% *v/v*, Panreac AppliChem, Barcelona, Spain; PAA), and the aliphatic diamine 3-(diethylamino)propylamine (≥ 99%, Sigma-Aldrich; DEAPA). As food and feed preservatives, we tested sodium chloride (VWR, Leuven, Belgium; NaCl), carvacrol (≥ 98%, Sigma-Aldrich; CAR), and L(+)-lactic acid (PanReac AppliChem; LA).

For all treatments, stationary-phase cultures were harvested by centrifugation (10,000 × *g*, 3 min) (MiniSpin, Eppendorf, Hamburg, Germany) and washed twice in PBS to remove residual growth medium. After a third centrifugation step, pellets were resuspended in the appropriate treatment medium. Working suspensions were prepared to achieve an initial concentration of approximately 3 × 10^7^ colony-forming units per mL (CFU/mL; *N*_*0*_) in the treatments.

Biocide treatments were conducted in sterile distilled water (10 mL in sterile flasks) to emulate disinfection protocols. Specifically, strains were exposed to 1.0 mg/L NaOCl (expressed as active chlorine) for 30 min, 0.5 mg/L PAA for 60 min, and 7.5 g/L DEAPA for 30 min. Food preservative treatments were performed in tryptone soya broth (Oxoid, Basingstoke, UK) supplemented with 0.6% (*w/v*) yeast extract (Oxoid; TSBYE) to simulate a high organic-matter environment relevant to food and feed matrices. Strains were exposed to 300 g/L NaCl for 96 h, 200 mg/L CAR for 60 min, and 10 g/L LA for 40 min.

Treatment times and intensities were selected based on preliminary experiments to achieve substantial but quantifiable inactivation of SeT. This was operationally defined as at least a 3-log_10_ reduction without reaching the assay quantification limit, represented as ≥ 5 log_10_ reduction, allowing comparison of strain-dependent responses. Since the tested compounds differ in antimicrobial activity and inactivation kinetics, different exposure times were required to obtain discriminatory treatment conditions. These conditions were therefore used as controlled comparative challenge tests and were not intended to directly reproduce a specific industrial disinfection or food-preservation protocol.

At defined time points during each treatment, aliquots were collected, immediately neutralized when required, serially diluted in PBS, and pour-plated in TSAYE. Excess of NaOCl and PAA (Merchel et al. 2021) were neutralized in PBS supplemented with sodium thiosulfate (10 g/L; Sigma-Aldrich), whereas DEAPA was neutralized in PBS supplemented with non-ionic surfactant polysorbate 80 (Tween 80, 30 g/L; Sigma-Aldrich). Untreated suspensions of each strain in the corresponding treatment medium, without antimicrobial compound, were used as controls to determine *N*_*0*_. Neutralizer controls were also included and confirmed that the neutralizing solutions alone showed no detectable bactericidal effect over the maximum treatment duration.

To minimize antimicrobial carry-over, undiluted samples were not plated; instead, only the 10^−1^ dilution and subsequent dilutions were plated, as preliminary experiments showed no residual antimicrobial activity from this dilution onwards under the tested conditions. Consequently, considering the initial concentration used in the treatments (approximately 3 × 10^7^ CFU/mL), the lowest plated dilution (10^−1^; 1 mL pour-plated), and the minimum countable threshold of 30 CFU per plate, the maximum reduction that could be reliably quantified was approximately 5 log_10_. Values reaching this threshold were therefore interpreted as reductions of ≥ 5 log_10_.

All treatments were conducted at room temperature (22 ± 1 °C, approximately) and with intermittent manual agitation. Viable counts were obtained by spread-plating on TSAYE and incubation at 37 °C for 24 h. Colony enumeration was performed using an automatic plate counter via image analysis (Analytical Measuring Systems, Protos, Cambridge, UK). Data were expressed as survival fractions (log_10_[*N*_*t*_/*N*_*0*_]) in time-course experiments or as log_10_ reductions (log_10_[*N*_*0*_/*N*_*t*_]) at selected treatment endpoints, where *N*_*0*_ corresponded to the initial untreated suspension of that same strain and *N*_*t*_ corresponded to the viable counts after *t* minutes of treatment. Logarithmic reductions were calculated relative to the untreated suspension (*N*_*0*_) of each strain. When viable counts fell below the quantification limit of the assay, reductions were represented at the corresponding limit for graphical purposes and interpreted as reductions of ≥ 5 log_10_. These values should therefore be considered censored values rather than exact reduction values. Survival risk was estimated from differences in log_10_ reductions, in which each log_10_ unit corresponds to a tenfold change in the survival risk.

*In vivo* virulence assessment using *Galleria mellonella* infection model.

The injection of pathogenic microorganisms into *G. mellonella* larvae is a widely used model for evaluating microbial virulence due to its operational simplicity, the absence of complex ethical requirements, and the presence of an innate immune response that shares relevant features with mammals (Ménard et al. 2021; Tsai et al. 2016). Wax moth larvae (*G. mellonella*) were purchased from a local commercial supplier (Reptilmadrid S.L., Madrid, Spain) and did not belong to a registered strain or laboratory-maintained line. Final-instar larvae weighing approximately 200–300 mg were stored in wood shavings, kept in the dark at room temperature, and used within 3 days of shipment. To minimize sampling bias, larvae were inspected before infection, and only healthy individuals without visible melanization, deformities, or signs of pupation were randomly selected.

To prepare injection suspensions, abundant biomass from several well-developed colonies was collected and transferred into sterile tubes containing PBS. Cells were centrifuged (3000 × *g*, 15 min) (Megafuge 1.0R, Heraeus, Hanau, Germany), the supernatant was discarded, and pellets were resuspended in sterile PBS. After washing, each suspension was standardized by adjusting optical density at 600 nm (OD_600_) to 0.5 McFarland (OD_600_ = 0.65–0.75) using a spectrophotometer (Libra S12, Biochrom Ltd, Cambridge, UK), corresponding to approximately 10^8^ CFU/mL (Farzana et al. 2018). Serial decimal dilutions in PBS were then prepared to obtain the final inocula used for injection.

Larvae were refrigerated for 30 min before injection to reduce activity. Based on prior tests (data not shown), each bacterial strain was injected at two concentration levels, approximately 10^4^ and 10^5^ CFU/mL (10 µL injected per larva). Inocula were confirmed by plate counts on TSAYE from the same suspensions used for injection. Injections were performed using sterile 1-mL syringes with 25-gauge needles (B. Braun, Melsungen, Germany) through the rear left proleg at an angle of 10–20° to access the hemocoel while minimizing tissue damage (Kay et al. 2019; Yang et al. 2017). A control group injected with 10-µL sterile PBS was included in all experiments.

Following injection, larvae were incubated at 37 °C (Tsai et al. 2016) and survival was monitored at 24, 48, and 72 h. Larvae were considered dead when no movement was observed upon gentle stimulation. Each condition was performed in triplicate with ten larvae per replicate. Mortality values over time were also used to calculate a pathogenicity index, weighting early deaths more heavily (Kay et al. 2019; Thomson et al. 2021).

### Statistical analysis

Statistical analyses and graph construction were performed using GraphPad Prism (GraphPad Software, San Diego, CA, USA). Lethality experiments were conducted in at least three independent biological replicates performed with different cultures on different days. Endpoint inactivation and time-course data were reported as mean ± standard deviation of log_10_ reductions. Normality was assessed using Kolmogorov–Smirnov and Shapiro–Wilk tests. Parametric data were analyzed using *t*-test or one-way ANOVA, as appropriate. When significant differences were detected, Tukey’s post hoc test was applied. Nonparametric data were analyzed using the Mann–Whitney *U* test or the Kruskal–Wallis’ test. The Mantel-Cox (log-rank) test was used to assess statistical differences between strains with multiple comparisons in constructed Kaplan–Meier survival curves of *G. mellonella*. Differences were considered statistically significant at *p* ≤ 0.05.

## Results

### Ciprofloxacin-resistant variants can display cross-protection to food-chain biocides

**Fig. 1:**
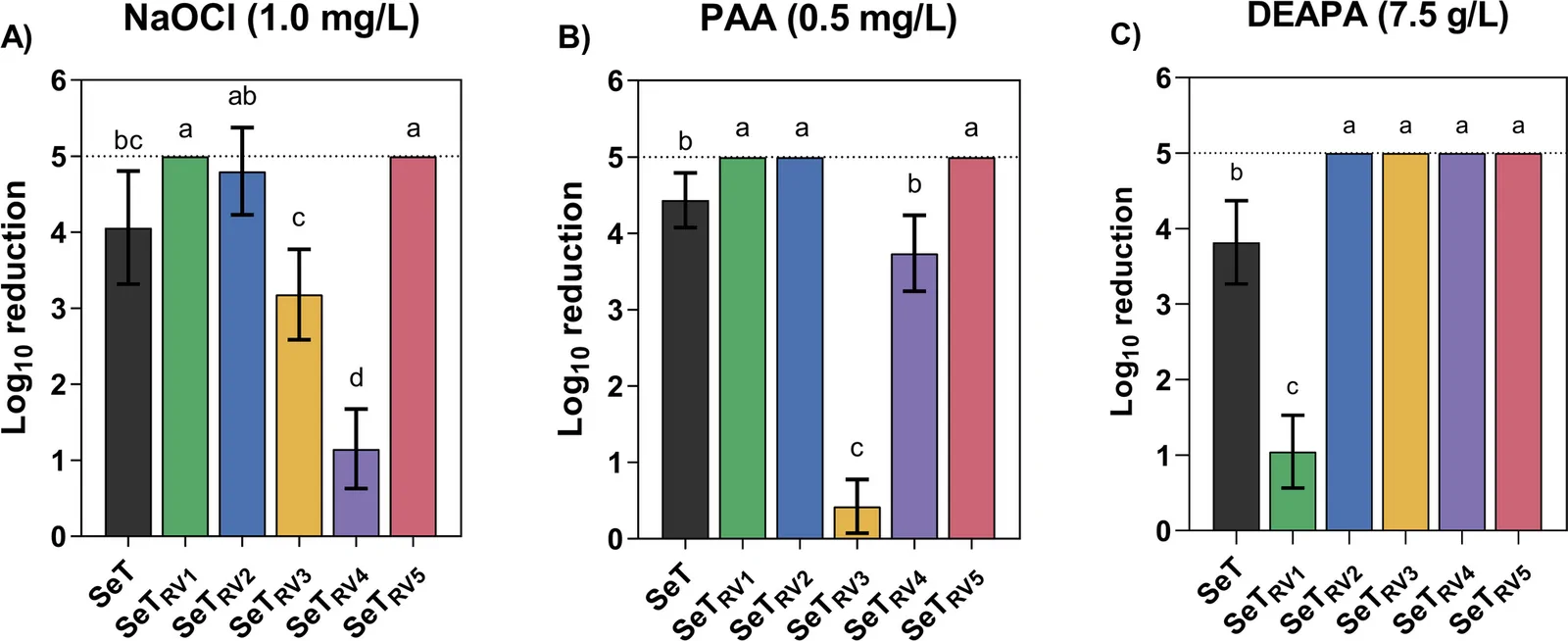
Log_10_ reductions (log_10_[*N*_*0*_/*N*_*t*_]) of *Salmonella enterica* subsp. *enterica* serovar Typhimurium str. LT2 parental strain (SeT) and its ciprofloxacin-resistant variants (SeT_RV1_-SeT_RV5_) after lethal exposure in distilled water to agri-food biocides: (**A**) sodium hypochlorite (NaOCl, 1.0 mg/L, 30 min); (**B**) peracetic acid (PAA, 0.5 mg/L, 60 min); and (**C**) 3-(diethylamino)propylamine (DEAPA, 7.5 g/L, 30 min). Bars represent mean ± standard deviation from at least 3 independent experiments. N_0_ ~ 3 × 10.^7^ CFU/mL. Horizontal dotted line represents the quantification limit. Values reaching the quantification limit represent reductions of ≥ 5 log_10_. Different letters indicate significant differences among strains within each panel (*p* ≤ 0.05)

We first assessed whether CIP resistance altered susceptibility to food-chain biocides by exposing the RVs to lethal treatments with NaOCl, PAA, and DEAPA (Fig. 1).

Overall, most RVs were as susceptible as, or more susceptible than, SeT under the tested conditions. However, this general trend differs from clear strain- and biocide-specific cross-protection phenotypes. For example, SeT_RV2_ showed no significant differences (*p* > 0.05) after NaOCl treatment (Fig. 1A), whereas SeT_RV5_ reached the quantification limit in all three biocide treatments, corresponding to reductions of ≥ 5 log_10_. Nonetheless, while NaOCl inactivated 4.1 log_10_ of SeT, SeT_RV4_ was reduced by only 1.2 log_10_, corresponding to ~ 1000-fold higher survival. SeT_RV3_ showed a stronger protective effect under PAA (Fig. 1B), with 0.4 log_10_ reduction compared with 4.4 for SeT (~ 10,000-fold higher survival). Finally, only SeT_RV1_ exhibited increased tolerance to DEAPA, with 1.1 log_10_ reduction compared with 3.85 for SeT (~ 1000-fold higher survival) (Fig. 1C).

### Ciprofloxacin-resistant variants can display cross-protection to food preservatives

**Fig. 2:**
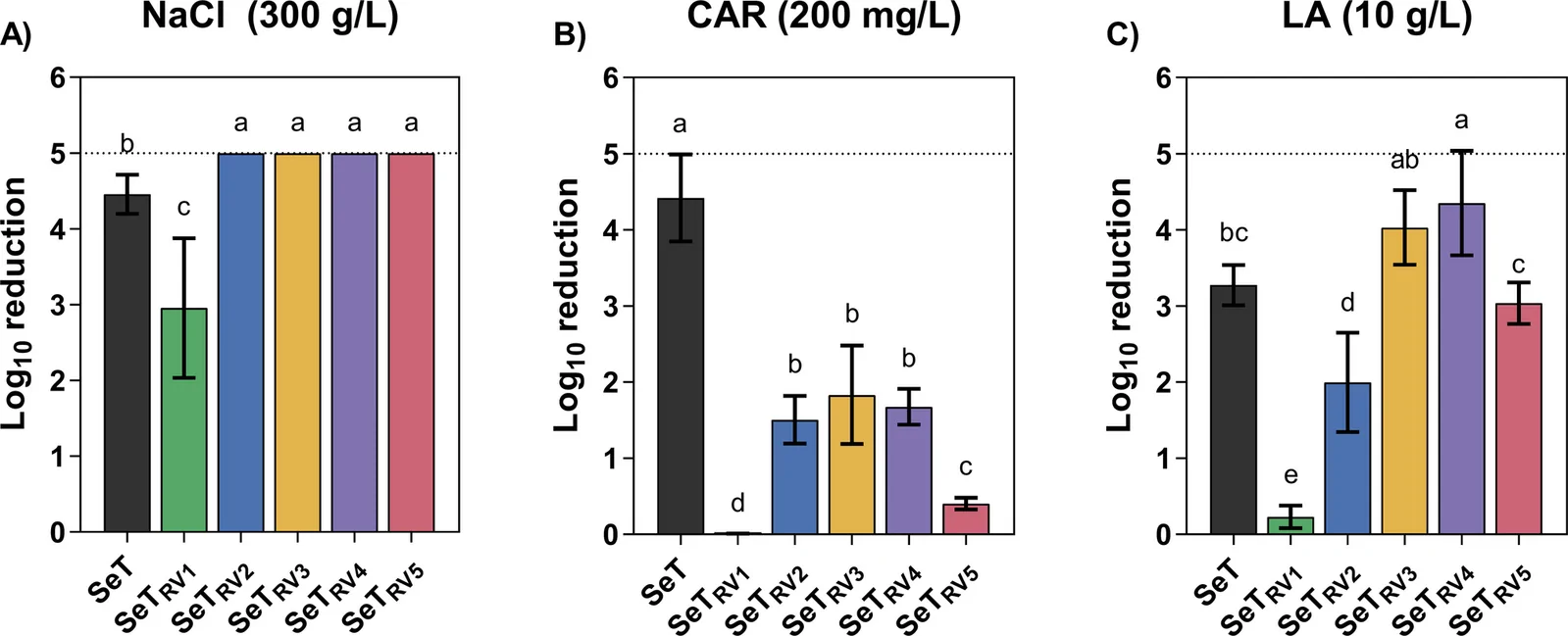
Log_10_ reductions (log_10_[*N*_*0*_/*N*_*t*_]) of *Salmonella enterica* subsp. *enterica* serovar Typhimurium str. LT2 parental strain (SeT) and its ciprofloxacin-resistant variants (SeT_RV1_-SeT_RV5_) after lethal exposure in TSBYE to food preservatives: (**A**) sodium chloride (NaCl, 300 g/L, 96 h); (**B**) carvacrol (CAR, 200 mg/L, 60 min); and (**C**) lactic acid (LA, 10 g/L, 40 min). Bars represent mean ± standard deviation from at least 3 independent experiments. N_0_ ~ 3 × 10.^7^ CFU/mL. Horizontal dotted line represents the quantification limit. Values reaching the quantification limit represent reductions of ≥ 5 log_10_. Different letters indicate significant differences among strains within each panel (*p* ≤ 0.05)

The response of the CIP-RVs to food preservatives was compound-dependent. While tolerance to NaCl and LA was observed only in specific variants, all CIP-RVs showed increased tolerance to CAR compared with SeT under the conditions tested (Fig. 2).

Under NaCl treatment, all RVs except SeT_RV1_ reached the quantification limit, indicating no cross-protection in these strains (Fig. 2A). In contrast, SeT_RV1_ showed clear cross-protection, with markedly reduced inactivation (~ 100-fold higher survival). Under CAR treatment, all CIP-RVs displayed increased tolerance, although the magnitude of the effect varied between strains (Fig. 2B). Among the RVs, SeT_RV1_ showed the largest increase in CAR tolerance, with no detectable inactivation under the conditions tested. Regarding LA, cross-protection was observed only in SeT_RV1_ and SeT_RV2_ (Fig. 2C): while SeT_RV2_ exhibited a slight but significant increase in tolerance (*p* ≤ 0.05), SeT_RV1_ showed a much stronger effect, with ~ 1000-fold higher survival compared with SeT.

### Differential contribution of mutations to cross-protection against selected farm-to-fork antimicrobial compounds

**Fig. 3:**
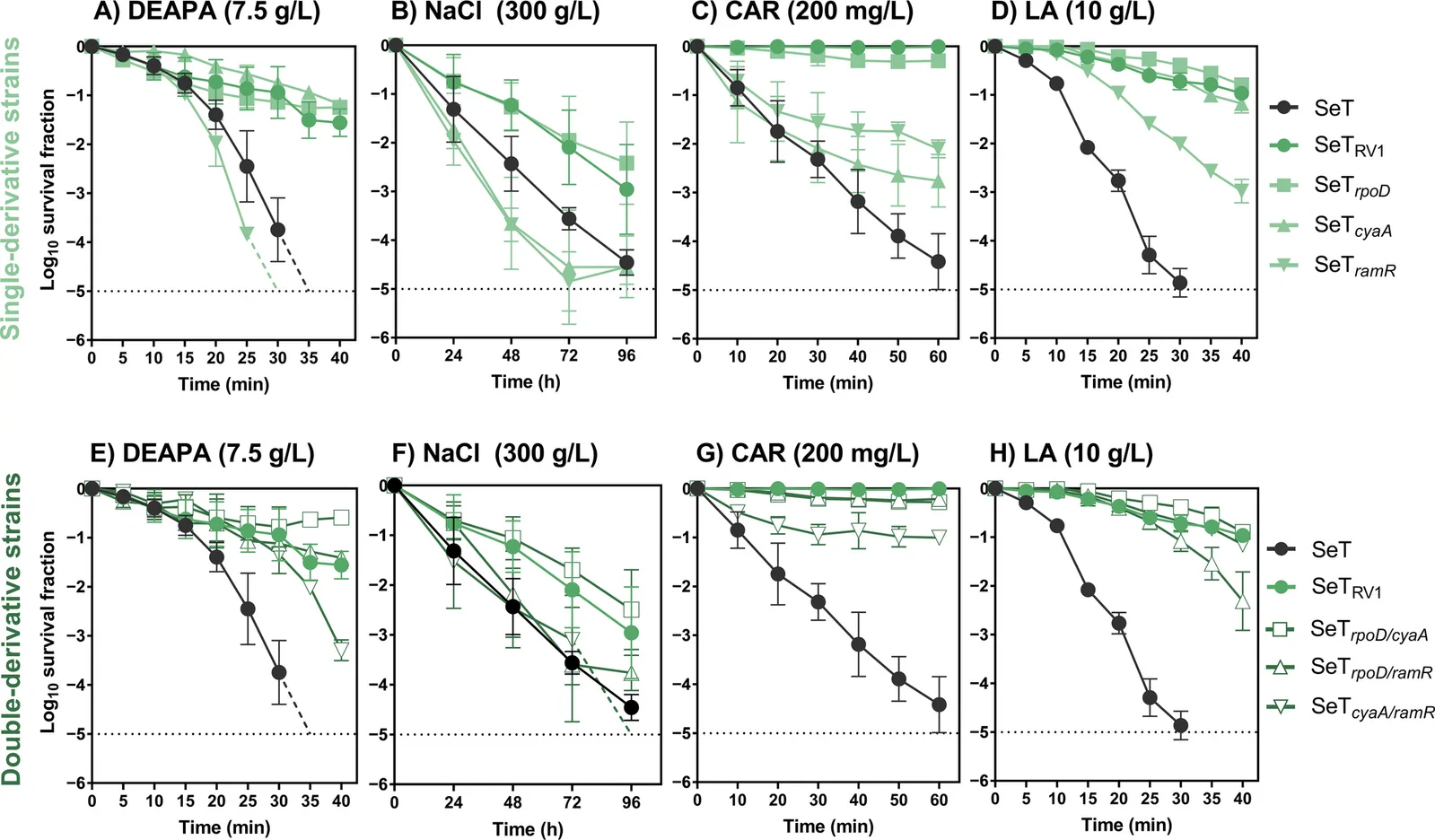
Survival fractions (log_10_[*N*_*t*_/*N*_*0*_]) over time of *Salmonella enterica* subsp. *enterica* serovar Typhimurium str. LT2 parental strain (SeT), its ciprofloxacin-resistant variant (SeT_RV1_), and SeT derivatives carrying SeT_RV1_-derived alleles as single-allele derivatives (**A**–**D** SeT_*rpoD*_, SeT_*cyaA*_, SeT_*ramR*_) or double-allele derivatives (**E**–**H** SeT_*rpoD/cyaA*_, SeT_*rpoD/ramR*_, SeT_*cyaA/ramR*_) during lethal exposure to, from left to right: 3-(diethylamino)propylamine (DEAPA 7.5 g/L, up to 40 min in distilled water); sodium chloride (NaCl, 300 g/L, up to 96 h in TSBYE); carvacrol (CAR, 200 mg/L, up to 60 min in TSBYE); and lactic acid (LA, 10 g/L, up to 40 min in TSBYE). Data represent mean ± standard deviation from at least 3 independent experiments. N_0_ ~ 3 × 10^7^ CFU/mL. Horizontal dotted line represents the quantification limit. Values reaching the quantification limit represent reductions of ≥ 5 log_10_

To identify which SeT_RV1_ mutations underpin broad cross-protection, we evaluated SeT derivatives carrying SeT_RV1_-derived alleles as single-allele derivatives (SeT_*rpoD*_, SeT_*cyaA*_, SeT_*ramR*_) and double-allele derivatives (SeT_*rpoD/cyaA*_, SeT_*rpoD/ramR*_, and SeT_*cyaA/ramR*_) under the same lethal treatments in which SeT_RV1_ showed increased tolerance (Fig. 3). Overall, distinct mutation-specific phenotypes were observed depending on both the antimicrobial compound evaluated (from left to right: DEAPA, NaCl, CAR, and LA) and the genetic background of the derivatives, including single-allele derivatives shown in the top panels (Fig. 3A–Fig. 3D) and double-allele derivatives shown in the bottom panels (Fig. 3E–Fig. 3H). In both cases, SeT and SeT_RV1_ are shown to facilitate comparison.

Compared with SeT, the *ramR* allele did not increase tolerance under DEAPA treatment (Fig. 3A) or NaCl exposure (Fig. 3B), whereas SeT_*ramR*_ showed increased survival under CAR (Fig. 3C) and LA (Fig. 3D). However, under LA treatment, this effect was more limited than that observed for other derivative strains. Similarly, the *cyaA* allele enhanced survival of SeT_*cyaA*_ under CAR and LA treatments, but also conferred increased tolerance to DEAPA. Notably, the *cyaA/ramR* double mutant (SeT_*cyaA/ramR*_) showed higher tolerance than either single mutant, particularly under CAR exposure (Fig. 3G) and compensating *ramR* costs against DEAPA (Fig. 3E).

In contrast, increased tolerance to DEAPA was only observed in strains carrying *rpoD* or *cyaA* (Fig. 3A). Interestingly, among all derivatives, SeT_*rpoD*_ displayed the broadest phenotype, with increased survival across all tested compounds. However, the *rpoD/ramR* combination against DEAPA (Fig. 3E), NaCl (Fig. 3F), and LA (Fig. 3H) did not result in an additive effect: while both mutations individually enhanced survival, their combination in SeT_*rpoD/ramR*_ only marginally improved tolerance compared with the *ramR* single mutant, suggesting a partial antagonistic interaction between *rpoD*- and *ramR*-mediated responses.

Regarding inactivation kinetics, DEAPA (Fig. 3A and Fig. 3E) and LA (Fig. 3D and Fig. 3H) showed an initial shoulder, defined here as a phase of delayed inactivation before a more rapid decrease in viable counts. In contrast, CAR and NaCl showed a more approximately log-linear decline, meaning that the inactivation rate was more constant when survival was represented on a logarithmic scale. In CAR treatments (Fig. 3C and Fig. 3G), several curves also showed tailing, defined as the persistence of a surviving fraction at longer exposure times, which may reflect more tolerant subpopulations or delayed activation of protective responses.

### Ciprofloxacin-resistant variants display divergent virulence phenotypes

**Fig. 4:**
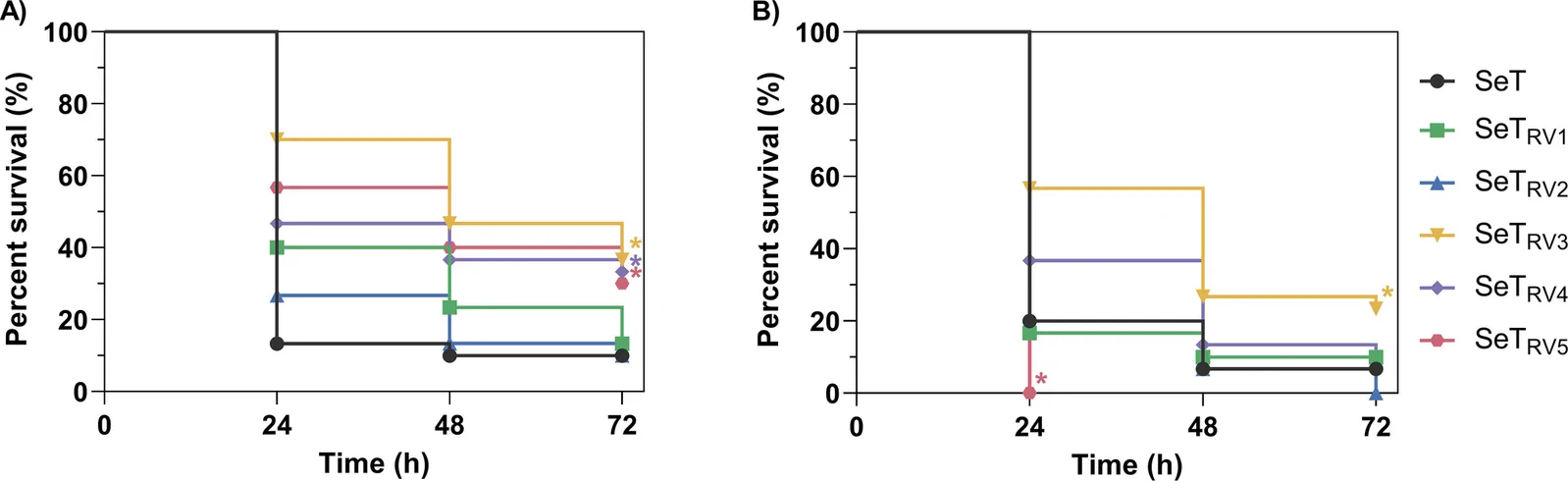
Kaplan–Meier survival curves of *Galleria mellonella* larvae after infection with *Salmonella enterica* subsp. *enterica* serovar Typhimurium str. LT2 parental strain (SeT) and its ciprofloxacin-resistant variants (SeT_RV1_, SeT_RV2_, SeT_RV3_, SeT_RV4_, and SeT_RV5_). Larvae were injected with bacterial suspensions at two inoculum levels: **A** 10^4^ CFU/mL and **B** 10.^5^ CFU/mL (10 µL per larva). Survival was monitored for 72 h. Curves show data from 3 biological replicates (*n* = 30 larvae per condition). Differences in survival were assessed using the Mantel-Cox (log-rank) test, and significant differences versus SeT (*p* ≤ 0.05) are indicated by an asterisk (*)

Following injection of SeT and the RVs into *G. mellonella* larvae, Kaplan–Meier survival curves were constructed to assess differences in larval survival as a proxy for virulence (Fig. 4). Each bacterial strain was injected at two microbial concentration levels: 10^4^ (Fig. 4A) and 10^5^ CFU/mL (Fig. 4B), approximately.

In larvae infected with SeT, survival decreased sharply within the first 24 h, reaching approximately 20% at both inoculum levels (Fig. 4A and Fig. 4B). Thereafter, survival changed only slightly, approaching a plateau until the end of the experiment. This suggests that, within the inoculum range tested, increasing bacterial concentration did not markedly change SeT-associated larval mortality (Fig. 4A and Fig. 4B).

Divergent *G. mellonella* pathogenicity patterns were observed among the RVs. SeT_RV2_ did not show statistically significant differences compared with SeT at either inoculum level concentration (*p* > 0.05; Fig. 4A and Fig. 4B). In contrast, SeT_RV3_ showed significantly reduced pathogenicity at both concentrations tested (*p* ≤ 0.05; Fig. 4A and Fig. 4B), SeT_RV4_ displayed a concentration-dependent response, with significantly reduced pathogenicity at the lower inoculum (10^4^ CFU/mL; *p* ≤ 0.05; Fig. 4A), whereas no differences were detected at 10^5^ CFU/mL (*p* > 0.05; Fig. 4B). SeT_RV5_ also showed a dose-dependent response, with reduced pathogenicity compared with SeT at 10^4^ CFU/mL (*p* ≤ 0.05; Fig. 4A), but increased pathogenicity at 10^5^ CFU/mL (*p* ≤ 0.05; Fig. 4B). Finally, SeT_RV1_ did not show significant differences compared with SeT at either inoculum level (*p* > 0.05; Fig. 4A and Fig. 4B).

## Discussion

As previously introduced, AMR can influence bacterial physiology beyond antibiotic survival, modulating tolerance to environmental stresses and antimicrobial interventions encountered along the food chain. In this work, our results revealed heterogeneous cross-protection patterns among the CIP-RVs. These findings indicate that cross-protection was not a uniform consequence of CIP resistance, but rather a strain- and compound-dependent phenotype arising from the interaction between the mutational background of each RV and the antimicrobial compound evaluated. This non-uniformity was particularly evident among the biocides tested (Fig. 1), where the response likely reflected the interaction between the specific mode of action of each compound and the genetic background of each RV.

Although NaOCl and PAA are both oxidizing biocides (Maillard and Pascoe 2024), their modes of action differ and may therefore be influenced by different physiological traits. PAA mainly causes oxidative damage to proteins, lipids, and nucleic acids, whereas NaOCl generates hypochlorous acid and chloramines that can affect multiple cellular targets, including metabolic processes (Aucinaite et al. 2025; Mohammadi 2008). Thus, the contrasting responses observed for SeT_RV3_ and SeT_RV4_ may reflect the interaction between these distinct modes of action and the specific mutational background of each RV. For example, SeT_RV3_ carries a mutation in *soxR*, a redox-sensing regulator that could influence oxidative-stress responses relevant to PAA exposure (Méndez et al. 2022), whereas SeT_RV4_ carries a *rpoC* mutation that may affect global transcriptional regulation and metabolic state, which could indirectly influence NaOCl tolerance. However, these links should be interpreted as plausible explanations rather than demonstrated causal relationships.

Beyond oxidizing biocides, evidence linking AMR-associated evolution with altered susceptibility to food-preservation compounds remains limited. However, previous studies have reported that mutations conferring AMR can alter susceptibility to chemically unrelated compounds. For example, induced meropenem, colistin, amoxicillin, and erythromycin resistance in *S*. Typhimurium has been associated with decreased sensitivity to CAR (García-Penas et al. 2026a; Tavares da Silva et al. 2025), and mutations in fatty acid biosynthesis genes (*fab*) have been linked to resistance to triclosan and isoniazid (Heath and Rock 2000﻿; Liu et al. 2024; Zhu et al. 2010). In line with this, the physiological alterations associated with CIP resistance, particularly those affecting membrane permeability, efflux activity, or global regulatory pathways (Azargun et al. 2020; Chang et al. 2021), may also explain enhanced bacterial responses to food-related antimicrobial compounds such as CAR or LA.

Although the RVs displayed heterogeneous and compound-specific responses, SeT_RV1_ showed the broadest cross-protection profile and was therefore selected for a more detailed analysis of the contribution of its mutations.

The SeT_RV1_-derived *ramR* allele increased survival of SeT_*ramR*_ compared with SeT during exposure to CAR and LA, but not under DEAPA or NaCl treatments. RamR is a transcriptional repressor of *ramA*, which regulates the AcrAB-TolC efflux system and other envelope-associated responses (Matsumoto et al. 2019; Holden and Webber 2020). In addition, changes in the RamR/RamA regulatory axis have previously been associated with adaptation to antibiotics and biocides in *S. enterica* (O’Regan et al. 2009; Wu-Chen et al. 2023). Thus, the phenotype observed for SeT_*ramR*_ is compatible with a possible contribution of efflux-related or envelope-associated changes to CAR and LA tolerance.

The SeT_RV1_-derived *cyaA* allele also increased survival under several chemical stresses, particularly DEAPA, CAR, and LA. *cyaA* encodes adenylate cyclase, which produces cAMP and influences CRP-dependent regulation of carbon metabolism and stress physiology (Görke and Stülke 2008). Therefore, the phenotypes observed in SeT_*cyaA*_ and in the *cyaA*-containing double derivatives suggest that changes in global metabolic regulation may contribute to survival under these treatments.

Among the tested single-allele derivatives, SeT_*rpoD*_ showed increased survival across all treatments evaluated. *rpoD* encodes the primary sigma factor σ^70^, which controls housekeeping transcription and may influence the balance between growth-associated and stress-associated transcriptional programmes. This interpretation is consistent with previous studies showing that changes in sigma-factor activity can affect stress-response allocation and multi-stress tolerance (Gao et al. 2016; Zhang et al. 2015; Park et al. 2024; Patten et al. 2004). The broad phenotype observed for SeT_*rpoD*_ therefore supports an important contribution of altered transcriptional regulation to the SeT_RV1_ response.

Overall, the derivative strains indicate that *ramR*, *cyaA*, and especially *rpoD* make important contributions to the broad SeT_RV1_ phenotype in a mutation- and compound-dependent manner. However, these results do not demonstrate that this combination is sufficient to fully reproduce the evolved variant, nor do they identify the precise molecular mechanisms involved. As efflux activity, cAMP levels, redox state, membrane properties, and gene expression were not directly assessed, these interpretations should be considered plausible functional links based on gene function, previous literature, and observed survival phenotypes, rather than experimentally validated mechanisms.

The remaining RVs (SeT_RV2_-SeT_RV5_) showed more heterogeneous and strain-specific phenotypes. The contrasting responses of SeT_RV3_ and SeT_RV4_ support the interpretation that cross-protection against oxidizing biocides depends on the specific adaptive trajectory rather than on CIP resistance itself. Their distinct mutational profiles suggest different physiological adaptations: NaOCl-derived chloramines can disrupt central metabolism (Zheng et al. 2024) supporting a possible role of metabolic state in NaOCl susceptibility, whereas PAA-mediated oxidative stress may be influenced by redox regulation and DNA-supercoiling-dependent transcriptional responses (Han et al. 2012; Martis et al. 2019; Méndez et al. 2022; Xie et al. 2024). These links remain plausible explanations rather than demonstrated mechanisms.

In contrast to the other compounds, CAR tolerance was recurrent among all CIP-RVs, although the magnitude of the effect varied substantially among variants. This suggests that CAR susceptibility may be particularly sensitive to physiological changes associated with CIP-driven evolution in this experimental system, in agreement with García-Penas et al. (2026a), who reported variant-dependent CAR cross-protection in *S.* Typhimurium RVs to antibiotics from different classes. However, this finding should not be generalized to all natural preservatives or resistant Gram-negative bacteria without broader testing. Alterations in the RamAR regulatory region in several CAR-tolerant RVs may contribute through envelope- or efflux-related responses, although SeT_RV2_ indicates that CAR tolerance cannot be explained solely by this locus. Other changes, such as *soxR* alterations, may also influence oxidative- or acid-stress responses relevant to phenolic compounds (Pagán et al. 2024b; Xie et al. 2024).

Overall, these results indicate that CIP-driven evolution can generate variants with broader tolerance profiles under defined laboratory conditions. However, the selection frequency and practical relevance of these phenotypes under real food-chain conditions remain to be established.

The *G. mellonella* assays further showed that CIP resistance did not have a uniform effect on pathogenicity in this model. Differences among RVs may reflect strain-specific fitness costs or regulatory changes associated with their distinct mutational backgrounds (Berdejo et al. 2024; Sobota et al. 2022). Importantly, SeT_RV1_ showed pathogenicity comparable to SeT, suggesting that its broad cross-protection phenotype did not coincide with a major loss of virulence-associated traits in this model, as also supported by the similarity in pathogenicity index values between SeT and SeT_RV1_ (Fig. S1). This is consistent with the broader view that resistance-associated mutations may reduce, preserve, or enhance virulence depending on the genetic background and infection model (Cepas and Soto 2020).

However, these observations should be interpreted as an initial indication of maintenance of virulence-associated pathogenicity in the *G. mellonella* model rather than definitive evidence of unchanged virulence in mammalian hosts. Although this model is useful for comparative virulence screening due to its ability to assess temperature-dependent virulence factors at 37 °C and the presence of an innate immune response sharing relevant features with mammals (Tsai et al. 2016; Ménard et al. 2021), it does not reproduce key aspects of *Salmonella* infection in mammals, including intestinal colonization, adaptive immunity, and host-specific physiological responses. Therefore, confirmation in more specific host models would be required to determine whether these RVs retain full pathogenic potential.

Whether CIP-RVs combine increased tolerance to farm-to-fork antimicrobial compounds with preserved virulence in this model, they could be more likely to persist through certain microbial control steps, potentially increasing contamination risk and consumer exposure. However, these phenotypes were highly heterogeneous among RVs and should not be interpreted as a generalized consequence of CIP resistance.

From a practical perspective, these findings may be relevant in food-chain environments where chemical antimicrobial interventions are applied, including sanitation with NaOCl, PAA, or DEAPA, lactic-acid-based carcass decontamination (Rodríguez-Melcón et al. 2017), plant-derived antimicrobials such as CAR, and salt-associated preservation hurdles. However, some concentrations and exposure times were selected to generate standardized, discriminatory stress conditions rather than to reproduce specific industrial protocols. Therefore, the findings should be interpreted as evidence of potential reduced susceptibility under defined challenge conditions, not as evidence that current sanitation procedures are ineffective. Further validation under realistic food-matrix conditions, organic load, and industrially relevant contact times is required.

Theoretically, selection of such RVs would be most plausible where *Salmonella* encounters fluoroquinolone pressure or antimicrobial residues, including animal production environments, manure, or contaminated food/feed by-products. Once selected, they could enter the food chain through colonized animals, raw materials, water, feed ingredients, or processing environments. However, this study did not assess occurrence, selection frequency, transmission, or persistence under real food-chain conditions, so these scenarios remain hypothetical and require further validation.

From a microbial risk assessment perspective, mathematical modelling of the behavior of such RVs could help capture uncertainties their potential persistence and transmission. Taken together, CIP-driven evolution generated variants with reduced susceptibility to farm-to-fork antimicrobial compounds without necessarily reducing pathogenicity in the *G. mellonella* model, underscoring the need to consider AMR-linked cross-protection in food-chain control strategies.

## Conclusions

The CIP-driven evolution in *S.* Typhimurium can reshape bacterial responses to food-chain antimicrobial compounds, but not as a uniform consequence of CIP resistance. Instead, cross-protection emerged as a heterogeneous, variant-, and compound-dependent phenotype, reflecting the interaction between antimicrobial mode of action and mutational background. SeT_RV1_ provided the clearest example of a broad cross-protection profile, and the analysis of derivative strains supported important contributions of *ramR*, *cyaA*, and especially *rpoD*, while also indicating that the complete evolved phenotype cannot be reduced to a single mutation or mechanism.

Importantly, altered susceptibility to food-chain antimicrobial compounds did not necessarily coincide with loss of virulence-associated pathogenicity in the *G. mellonella* model. Overall, these findings suggest that AMR-associated phenotypic changes may influence bacterial persistence against selected food-chain antimicrobial interventions. However, their practical relevance should be further assessed under realistic food-matrix, sanitation, and host-associated conditions before drawing conclusions for specific industrial settings or risk assessment scenarios.

## Supplementary Information

Below is the link to the electronic supplementary material.

Supplementary file1 (PDF 185 kb)

## Data Availability

Sequencing data associated with the resistant variants analyzed in this study were previously deposited in the NCBI Sequence Read Archive under BioProject accession number PRJNA1044031. The corresponding sample accession numbers were reported by Campillo et al. (2025; DOI: https://doi.org/10.1016/j.foodres.2025.116330). Additional experimental data generated during this study will be made available from the corresponding author upon request.
