# Supplementary material for: Reduced efficacy of food-chain biocides and preservatives against ciprofloxacin-resistant Salmonella Typhimurium
Source: Appl Microbiol Biotechnol. 2026 Jul 28;110:319. doi: 10.1007/s00253-026-13973-0 (PMC13694529; doi:10.1007/s00253-026-13973-0)
Supplement: Supplementary file 1 [file 253_2026_13973_MOESM1_ESM.pdf]

Full title: Reduced efficacy of food-chain biocides and preservatives against ciprofloxacin-resistant *Salmonella*

Journal: Applied Microbiology and Biotechnology

Authors: Raúl Campillo[1], Ivo García-Penas[1], Celia Pardos[1], Patricia Gimeno[1], Daniel Berdejo[1], Rafael Pagán[1], and Diego García-Gonzalo[1]*

Author affiliations:

[1]Departamento de Producción Animal y Ciencia de los Alimentos, Facultad de Veterinaria, Instituto Agroalimentario de Aragón-IA2 (Universidad de Zaragoza-CITA), Zaragoza, Spain

Contact information for corresponding author:

*Dr. Diego García Gonzalo

Diego.Garcia@unizar.es

Departamento de Producción Animal y Ciencia de los Alimentos, Facultad de Veterinaria, Instituto Agroalimentario de Aragón-IA2 (Universidad de Zaragoza-CITA)

C/ Miguel Servet 177, 50013, Zaragoza, Spain

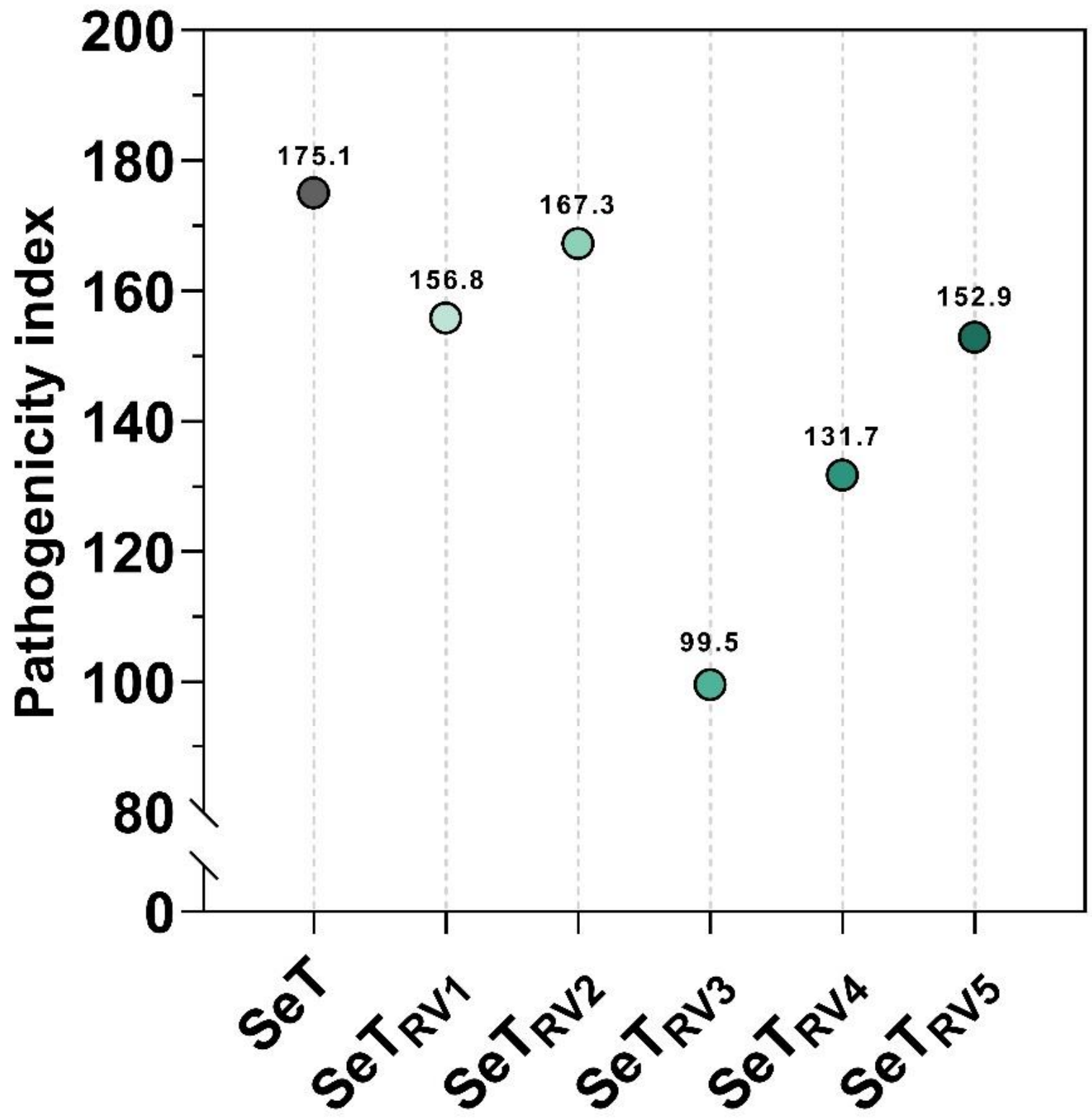

Figure S1. Pathogenicity index values in the *Galleria mellonella* infection model for *Salmonella enterica* subsp. *enterica* serovar Typhimurium str. LT2 parental strain (SeT) and its ciprofloxacin-resistant variants (SeT_{RV1}, SeT_{RV2}, SeT_{RV3}, SeT_{RV4}, and SeT_{RV5}).
